# Molecular Therapies for Myotonic Dystrophy Type 1: From Small Drugs to Gene Editing

**DOI:** 10.3390/ijms23094622

**Published:** 2022-04-21

**Authors:** Mariapaola Izzo, Jonathan Battistini, Claudia Provenzano, Fabio Martelli, Beatrice Cardinali, Germana Falcone

**Affiliations:** 1 Institute of Biochemistry and Cell Biology, National Research Council, Monterotondo, 00015 Rome, Italy; mariapaola.izzo@ibbc.cnr.it (M.I.); jonathan.battistini@ibbc.cnr.it (J.B.); claudia.provenzano@cnr.it (C.P.); 2 Molecular Cardiology Laboratory, IRCCS Policlinico San Donato, San Donato Milanese, 20097 Milan, Italy; fabio.martelli@grupposandonato.it

**Keywords:** myotonic dystrophy, trinucleotide-expansion disease, DM1 mice, antisense oligonucleotides, molecular therapy, gene editing

## Abstract

Myotonic dystrophy type 1 (DM1) is the most common muscular dystrophy affecting many different body tissues, predominantly skeletal and cardiac muscles and the central nervous system. The expansion of CTG repeats in the DM1 protein-kinase (*DMPK*) gene is the genetic cause of the disease. The pathogenetic mechanisms are mainly mediated by the production of a toxic expanded CUG transcript from the *DMPK* gene. With the availability of new knowledge, disease models, and technical tools, much progress has been made in the discovery of altered pathways and in the potential of therapeutic intervention, making the path to the clinic a closer reality. In this review, we describe and discuss the molecular therapeutic strategies for DM1, which are designed to directly target the CTG genomic tract, the expanded CUG transcript or downstream signaling molecules.

## 1. Introduction

Myotonic dystrophy type 1 (DM1) is caused by an unstable expanded CTG repeat located within the 3′ untranslated region (3′ UTR) of the *DMPK* gene. The molecular mechanisms of DM1 are mainly the consequence of accumulation of mutant *DMPK* transcripts into ribonuclear foci leading to the impairment of alternative splicing and normal gene expression. Cell and animal models of DM1 have been crucial to providing insight into disease mechanisms and to revealing new therapeutic targets. This review outlines the clinical features and pathogenetic mechanisms of DM1 and provides an updated description of the many different therapeutic approaches for DM1, ranging from antisense- and small-drug-mediated strategies to gene therapy, with a focus on those under preclinical and clinical evaluation.

## 2. Myotonic Dystrophy Type 1: Clinical Features and Pathogenetic Mechanisms

**Figure 1:**
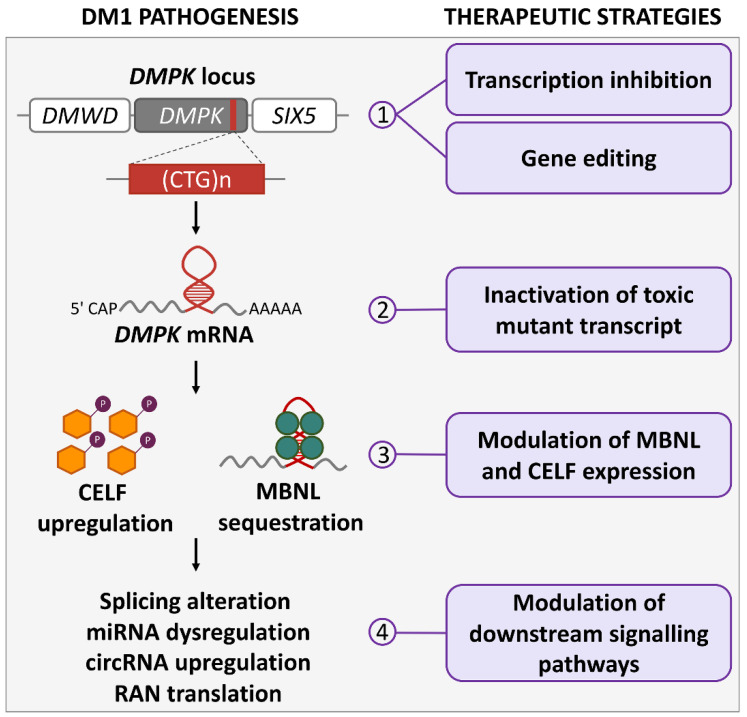
DM1 pathogenetic mechanisms and therapeutic strategies. The actions of molecular therapies for DM1 at different pathogenetic levels are illustrated: (1) at DMPK gene, drugs can inhibit CTG-repeat transcription and induce repeat contraction; ZFN, TALEN or CRISPR/Cas9 nucleases can modify gene sequence by inducing CTG-repeat contractions or deletions, or by inserting premature polyadenylation signals; (2) mutated DMPK mRNA can be functionally inactivated by drugs inducing degradation or binding to CUG repeats; (3) MBNL can be released from CUG repeats by disruption of MBNL:CUG interaction through competitive binding, and CELF levels can be regulated by protein kinase C and glycogen synthase kinase 3β; (4) altered signaling pathways downstream of DMPK transcript can be rescued by modulation of splicing and miRNAs; circRNAs and RAN translation could also be targets of future therapies.

DM1 is the most common dystrophy in adults, having an estimated worldwide prevalence of 1:20,000, with a recent report of 4.76:10,000 for *DMPK* CTG expansion ≥50 CTG repeats in a newborn screening program in New York State, USA [1]. Clinical features of DM1 (Steinert’s disease, OMIM# 160900) include muscle weakness, dysphagia, neuromuscular respiratory insufficiency, cardiac complications and cognitive, intellectual or behavioral impairment as well as sleep disorders. In the most severe forms, life quality and expectancy are seriously compromised [2,3,4]. DM1 results from CTG-repeat expansions in the 3′ UTR of the *DMPK* gene on chromosome 19 [5,6]. The disease severity and age of onset are broadly correlated with the number of CTG repeats, with the highest (over 750) in the congenital form, while in non-affected individuals the number of repeats is up to 35 [2]. The number of repeats is usually unstable and tends to increase in some body tissues during lifetime (somatic instability) as well as in successive generations, leading to the phenomenon called “anticipation”, where children of DM1 patients have a higher repeat number and more severe phenotypes compared to their parents [7]. Interestingly, in DM1 families with variant repeats, where GGC, CCG and CTC interruptions are present within the CTG-repeat array, the repeats are stabilized and the disease phenotypes are milder [8,9]. Several pathogenic mechanisms likely contribute to disease in DM1 (Figure 1) [4,10]. At the DNA level, epigenetic modifications may impact the development or severity of the phenotype in DM1 patients [11]. In DM1 patient-derived cells and in a DM1 mouse model, the hairpin-like structures of the repeats can induce chromatin changes, such as CpG methylation, resulting in haploinsufficiency of *DMPK* and neighboring genes, or cause replication-fork stalling during DNA duplication, leading to cell stress [12,13,14]. Large experimental evidence supports the hypothesis of an RNA gain-of-function mechanism of the mutated *DMPK* transcript. CUG-containing RNAs sequester crucial nuclear factors of the muscleblind-like (MBNL) family into ribonuclear foci, thus preventing their normal functions that are mainly associated with the regulation of alternative splicing [15]. Splicing regulation is required for the proper development and maintenance of tissues in which the *DMPK* gene is highly expressed, such as in muscle and the nervous system [16]. The MBNL family and the CUGBP Elav-like family (CELF) are among the most important splicing regulators in skeletal and cardiac muscle, and act antagonistically on several pre-mRNA targets [17,18]. Nuclear retention of MBNL proteins in nuclear foci prevents pre-mRNA processing and export to the cytoplasm, leading to a decrease in protein translation, and the loss of functional MBNL1 is accompanied by CELF1 upregulation [19]. The increase in CELF1 levels is induced by protein-kinase-C (PKC)-mediated hyperphosphorylation, leading to protein stabilization [20]. Both sense and antisense repeated RNAs have been shown to contribute to the clinical phenotype of nucleotide-expansion diseases [21]. An antisense transcript emanating from the *DMPK*-adjacent *SIX5* regulatory region spanning the CTG expansion was first identified in DM1 patient-derived cells. The transcript was shown to be converted into siRNAs, which are able to recruit DNA and histone methyltransferases, leading to heterochromatin formation [22]. Interestingly, in transgenic mice carrying the human *DMPK* locus, in addition to CUG-containing transcripts, CAG-containing transcripts were also found to form distinct ribonuclear foci [23].

Other mechanisms involved in DM1 pathogenesis are repeat-associated non-ATG (RAN) translation (reviewed in [24]), which results in the production of toxic protein aggregates containing polyglutamine from antisense CAG-repeated transcripts [25,26], microRNA (miRNA) dysregulation [27,28,29,30], and upregulation of circular RNA (circRNA) expression [31,32,33]. In addition to the ones described above, other signaling cascades are affected by the toxic *DMPK* RNA and may play important roles in DM1 pathogenesis. For example, MBNL and CELF regulators, besides being key splicing regulators, are likely involved in cytoplasmic pathogenetic processes altering proteostasis and sarcomere structure (reviewed in [34]). Omics studies have added new information to the previous knowledge, revealing several alterations in gene expression, alternative splicing, CpG methylation and proteins levels that potentially contribute to DM1 pathogenesis. In perspective, these new approaches can be crucial to evaluate the degree of therapeutic rescue and the off-target effects of drug candidates (reviewed in [35]).

## 3. DM1 Cell and Animal Models

In vitro models of DM1 have greatly contributed to clarifying the pathogenetic mechanisms of the disease. Among these, there are engineered cell lines with CTG repeats of different lengths inserted in minigenes [36,37], DM patient-derived primary myoblasts, immortalized myoblasts or MYOD1-converted fibroblasts [38,39,40,41], and embryonic stem cells [42]. Additionally, induced pluripotent stem cells (iPSCs) and iPSC-derived distinct cell types were used to study tissue-specific DM1 pathological alterations [43,44,45,46]. Recently, the first 3D in vitro human-muscle model of DM1 was developed by encapsulating patient-derived MYOD1-converted fibroblasts in hydrogels [47]. All of these cell models reproduce molecular alterations typical of DM1 and have been very useful for discovering crucial molecules and cellular pathways involved in the disease and for testing therapeutic strategies.

*Drosophila* models have also been used by several groups to study DM1. Interrupted CTG repeats of various lengths driven by either constitutive or inducible promoters were expressed in flies and DM1-related molecular as well as phenotypic alterations were observed in flies carrying more than 480 repeats [48,49,50]. Although DM1 modeling is complicated by the multifaceted impact of the DM1 mutation, many DM1 mouse models have been generated over time through the silencing of the *Dmpk* gene or the *Mbnl* family genes; alternatively, mouse models expressing CELF proteins or toxic CTG repeats in various tissues were produced, in order to mimic the different aspects of the disease and to discover therapeutic molecules (Table 1). It is unclear whether the *DMPK* haploinsufficiency observed in DM1 patients may affect functions of the tissues in which the gene is normally highly expressed, such as muscles and the central nervous system (CNS). To address this question, different *Dmpk*-knockout (KO) mouse models have been generated and characterized through the years with different results. Initial reports on *Dmpk*-KO mice described cardiac conduction defects [51] and mild myopathy [52]. Since these mice models were characterized by a mixed genetic background possibly leading to confounding effects, more recently a *Dmpk*-KO model backcrossed to two different pure genetic backgrounds was generated. This model did not confirm the previous observations, but showed that *Dmpk* gene deletion does not compromise cardiac or skeletal-muscle function [53]. Additionally, *DMPK* transcript silencing through antisense oligonucleotides (ASOs) was well tolerated in mice, rats and monkeys [54]. These findings suggest that reduction in *DMPK* expression should not be a prominent cause of the disease. Given the crucial regulatory role of MBNL proteins in DM1, different *Mbnl* KO models were generated to elucidate the role of each MBNL protein in the disease. *Mbnl1* and *Mbnl2* loss of function resulted in muscular and CNS symptom manifestation, respectively [55,56,57], while *Mbnl3* KO caused a progressive delay in muscle regeneration and embryonic muscle differentiation abnormalities, in agreement with their expression profiles during development [58]. Mice with double *Mbnl1/Mbnl2* KO or *Mbnl1/Mbnl3* KO exhibited more severe phenotypes compared with the single KO [59,60] and the triple *Mbnl1/2/3* KO in muscle tissues recapitulated the severe phenotype observed in congenital DM1, in both newborn and adult mice [61], supporting the idea of a prominent role of MBNL proteins and alternative splicing dysregulation in DM1 pathogenesis. To determine the role of CELF proteins in DM1 pathogenesis, mouse models overexpressing CELF1 and CELF2 in skeletal and/or cardiac muscle were generated [62,63,64,65]. Overexpression of CELF1 was shown to reproduce DM1-associated histopathological and functional changes [63]. Notably, CELF1/2 overexpressing mouse models have revealed a strong pattern of antagonistic regulation of mRNA levels by CELF and MBNL proteins through competitive binding to 3′ UTR regions [64] (Table 1A). 

Based on the assumption that the expanded *DMPK* transcript is the main cause of DM1 disease, many different mouse models expressing expanded CUG transcripts either ubiquitously or in specific tissues were generated to model the disease mechanisms (Table 1B). The multisystemic impact of CUG expansions is well recapitulated in DM200, DM300 and in DMSXL transgenic mice carrying the 3′ UTR portion or the entire human *DMPK* gene with CTG repeats of different lengths, the phenotype being more severe in mice with larger expansion [23,68,71]. In DM300 and DMSXL mice, transgene expression resulted in the accumulation of ribonuclear foci in various tissues and in the development of muscle weakness, behavioral abnormalities, growth retardation and perinatal mortality [23,71,72,73,74]. Recently, a mouse model constitutively expressing CTG repeats within the *DMPK* context was generated, which exhibited particularly high CUG expression in the heart (LC15). These mice reproduced DM1-like cardiac defects [77]. Skeletal-muscle-specific, heart-specific and brain-specific DM1-like features have been reproduced in mouse models expressing the repeat expansion in the respective tissues, either constitutively (HSA^LR^) [76] or inducibly (EpA960 and TREDT960I mice strains) [75,78,79,80,81,82,83]. The tissue-specific phenotypes are usually strong and suitable for testing therapeutic molecules. However, at the same time, they do not recapitulate the multisystemic DM1 phenotype. In the inducible models, CTG repeats interrupted with stretches of CTCGA have been inserted in a portion of the *DMPK* human transgene. Interrupted repeats have the advantage of being more stable than CTG repeats, but may not exactly reproduce the human DM1 disease condition. Each of these mouse models exhibits advantages and limitations mostly depending on the temporal and spatial control of the transgene expression (detailed in Table 1). Taken together, transgenic mouse models have been crucial to understanding DM1 pathogenetic mechanisms and to testing therapeutic approaches.

## 4. Molecular Therapies Acting on *DMPK* Gene Expression

A number of therapeutic approaches have been tested for DM1, and some of them are in the preclinical stage of development in animal models and more recently in human clinical trials [84]. They have been designed to specifically target the mutant allele, its RNA product, or its downstream signaling pathways (Figure 1). Currently, the small-molecule approach is one of the preferred pharmacological strategies to treat diseases due to its robustness and cost-effectiveness. In the last few years, some of these compounds have shown significant activity against DM1 pathogenetic repeats through various mechanisms such as the inhibition of DNA transcription by binding CTG repeats, degradation of the toxic mutant transcript, release of MBNL from the ribonuclear foci through inhibition of MBNL:CUG transcript interaction, and lastly modulation of signaling pathways of downstream repeated RNA expression [85].

### 4.1. Induction of Repeat Contractions and Transcription Inhibition at DMPK Gene

A variety of approaches have been used in past and recent years to identify factors that can modify the instability of CAG/CTG repeats. These include reagents that directly interact with the repeat tract and DNA-repair proteins that act in pathways to enhance or suppress instability. Certain DNA-damaging agents, including UV light, alkylating and intercalating drugs, and anticancer agents such as cisplatin and X-rays were shown to increase the rate of deletion or induce suppression of somatic expansion of CTG/CAG repeats [86,87]. Other drugs such as caffein and DNA-replication inhibitors, on the contrary, increased the rate of expansion [86,88,89]. These pioneer studies have demonstrated that exposure to exogenously added compounds can specifically alter the genetic instability of the expanded CTG tract at the DM1 chromosomal locus in patient cells. In addition, they have highlighted that interference with DNA-replication and DNA-repair mechanisms is crucial to determining repeat expansion or contraction. An updated overview of modifiers of CAG/CTG-repeat instability in mammalian models can be found in [90]. Unfortunately, most of the tested drugs are effective at doses that are not therapeutically relevant and often lack sufficient specificity. Among the small molecules, the compound naphthyridine-azaquinolone (NA) has recently been shown to directly engage the repeat tract at the DNA level. NA can bind to slipped CAG DNA intermediates of expansion mutations of Huntington disease (HD) and efficiently induces repeat contraction and/or prevent expansion in HD patient cells and in the HD mouse model. NA specifically binds to CAG but not to CTG repeats and induces contractions only in the expanded allele in a transcription-dependent but replication-independent fashion [91]. Interestingly, an ASO-binding CUG-expanded RNA was also shown to reduce repeat instability in DM1 cell and mouse models, although the mechanism is not known [92].

Transcription elongation is a promising therapeutic target for repeat-expansion mutations including CTG repeats. For example, the transcription elongation factor Spt4 was shown to be required for transcription of expanded repeats [93]. Silencing of its human ortholog *SUPT4H1* inhibits the expression of expanded repeats in cultured cells derived from patients affected by repeat-expansion diseases [94,95]. Since *SUPT4H1* is involved in transcription of different types of repeats in both sense and antisense directions, this gene may be considered as a shared therapeutic target for repeat-expansion diseases. The antimicrobial drug pentamidine and related antibiotics were shown to bind CTG/CAG repeats, reduce the expression of expanded CUG RNA and rescue splicing defects in Hela DM1 cells expressing CTG repeats and in HLA^LR^ mice. However, toxicity was observed in treated mice, possibly due to insufficient binding specificity [96]. Additionally, actinomycin D, a DNA intercalator that preferentially inserts at GpC dinucleotides, was reported to reduce the expression of expanded CUG RNA at doses below those necessary for general transcription inhibition [97]. Selective modulation of toxic CUG RNA by transcription inhibition was recently obtained by using microtubule inhibitors such as colchicine, possibly via regulation of chromosome dynamics by microtubules through the linker-of-nucleoskeleton-and-cytoskeleton (LINC) complex [98].

### 4.2. Targeting Expanded CUG RNA 

In the last decade, a number of therapeutic approaches aimed at neutralizing the toxic effect of CUG expansion in DM1 have been developed. The properties and delivery strategies of nucleic-acid-based therapeutic molecules are summarized in Table 2. A widely studied group of drugs belongs to the class of ASOs [99]. Since unmodified ASOs can be degraded by nucleases in blood serum and eliminated via renal filtration [100], chemical modifications of ASOs have been introduced in order to increase the stability and efficacy of in vivo applications. Thanks to these features, modified ASOs can be directly administered without carriers, thus reducing the manufacturing costs [101]. Common changes to ASOs include backbone modifications (phosphorothioate and phosphorodiamidate, instead of phosphodiester) and/or sugar-ring modifications (2′-O-methyl (2′OMe), 2′-O-methoxyethyl (MOE), 2′,4′ carbon link (LNA, locked nucleic acid), 2′,4′ constrained ethyl (c-Et) and morpholino rings (PMO)) [99,102]. ASOs were designed with two different aims: (i) to block MBNL1 interaction by binding to CUG repeats or (ii) to induce the degradation of CUG-expansion-containing *DMPK* mRNA. 

#### 4.2.1. ASOs Blocking MBNL1/2 Binding

Different types of molecules with structures that are incompatible with RNase H degradation and that target CUG repeats to block interaction with MBNL1 have been used: PMO-CAG25 [104], 2′-OMe-CAG ASOs [103,126], LNA-CAG mixmers [92], all-LNA-CAG ASOs [105]. The effects of these molecules have been tested following intramuscular injection in either an HSA^LR^ mouse model or in mice expressing a full *DMPK* transgene containing 500 triplets derived from the DM300 mouse line [103]. In all cases, a reduction in the number of foci, redistribution of MBNL1 protein and efficient correction of abnormal RNA splicing were obtained [103,104,105]. In particular, in the HSA^LR^ model recovery of the alternative splicing of the muscle chloride-channel (*Clcn1*) transcript, which is involved in myotonia, was associated to a marked reduction in the defect [104]. Interestingly, a PMO-ASO-mediated approach was applied to correct defective splicing of *Clcn1* and led to amelioration of myotonia in HSA^LR^ [127]. Additionally, intramuscular injection of an optimized ASO sequence targeting *Clcn1* pre-mRNA, followed by ultrasound-mediated bubble liposome exposure, proved to be an effective strategy for ASO delivery into muscle tissues [128]. The penetration of ASOs in DM1 muscle tissue is challenging since, unlike in other muscle diseases, the fiber membrane integrity is not compromised [129]. Delivery strategies have been progressively adapted to the properties of the therapeutic molecules and of the target cell/tissue to be reached. PMO-CAG molecules have been conjugated to cell-penetrating peptides (PPMO-B, PPMO-K and Pip6a-PMO) and administered systemically. Besides foci reduction and splicing correction, myotonia was completely abolished in HSA^LR^ mice [106,107]. Recently, new relatively short miniPEG-γ peptide nucleic-acid probes, two triplet repeats in length, for greater ease of cellular delivery, have been described to discriminate the pathogenic CUG-expanded transcript from the wild-type transcript [109]. These molecules are currently undergoing preclinical testing (NT-0200) in the HSA^LR^ model [108]. Although designed to block CUG repeats and prevent MBNL1 binding without altering RNA target levels, modified ASOs were also shown to induce transcript degradation via an RNase-H-independent pathway [103,104,105]. A general observation concerning ASOs that hybridize directly with the repeated CUG sequence is that, since DM1 patients have a highly variable number of repeated sequences, personalized doses should be considered in order to neutralize the toxic CUG expansion. 

#### 4.2.2. ASOs Silencing Expanded CUG Transcript

The therapeutic antisense molecules designed to induce a reduction in CUG-expansion-containing *DMPK* mRNA exploit the RNase-H-degradation pathway. To allow RNase-H binding and activity, a gap of unmodified DNA nucleotides is added in between the modified 2′-OMe, 2′-MOE, c-Et or LNA nucleotides placed at both ends (gapmer) [102]. The gapmers target regions downstream or upstream of the CTG repeats [54,70,110,111,112,130], except those used by Lee and colleagues that directly targeted the repeat sequence [113]. The gapmers, delivered either locally in skeletal muscles or systemically via subcutaneous injection in different mouse models and nonhuman primates, induce the degradation of the target mRNA, foci number reduction, splicing correction [54,70,110,111,112,113,130], amelioration of myotonia, muscle strength and fatigue symptoms [54,111,112,130], and improvement of cardiac conduction defects [70]. Recently, a c-Et gapmer was delivered via intracerebroventricular injection in DMSXL mice brains where it efficiently reduced *DMPK* mRNA expression and corrected behavioral abnormalities in homozygous mice [110]. IONIS-DMPKRx, a c-Et-modified version of a MOE gapmer previously studied [112], was used in a clinical trial (NCT02312011) for the treatment of DM1 [114,131]. However, since the drug levels in muscle tissue were not sufficient to obtain the desired therapeutic benefit, the trial was interrupted [132]. In an effort to increase potency and drug uptake into target tissues [133], palmitoyl-conjugated c-Et gapmers have been used to enhance delivery to skeletal and heart muscle in preclinical studies [69,134,135]. 

#### 4.2.3. Other Strategies

In addition to ASO approaches, other strategies for targeting CUG expansion have been tried. Engineered hU7-snRNAs containing a poly-CAG antisense sequence were used to induce the degradation of the expanded CUG repeats of mutant *DMPK* transcripts in DM1 patient-derived cells [136]. RNA interference (RNAi) mediated by either intramuscular injection of siRNAs or systemic delivery of miRNA based RNAi hairpins was induced in HSA^LR^ mice, leading to the downregulation of the toxic transcript and significant reversal of DM1-like hallmarks [118,137]. Recently, therapeutic siRNAs conjugated to an antibody against the transferrin receptor 1 (AOC 1001), targeting the *DMPK* transcript, have shown efficient uptake in skeletal- and cardiac-muscle tissues. A clinical trial (NCT05027269) using these modified oligonucleotides is ongoing [119].

### 4.3. Targeting MBNL1/2

Due to the limitation of ASO delivery to all body tissues, particularly to the CNS, alternative approaches have been investigated in order to identify small molecules that upregulate MBNL proteins or inhibit the MBNL:CUG-repeat interaction and disperse RNA foci. In order to increase the functional pool of MBNL in DM1 cells/tissues and alleviate pathogenesis of DM1 by the reversal of aberrant alternative splicing, the expression of MBNL1/2proteins has been increased by using nonsteroidal anti-inflammatory drugs such as phenylbutazone (PBZ) and ketoprofen [138], and antiautophagic drugs such as chloroquine [139], or by delivery of recombinant-adeno-associated-virus (rAAV) vectors carrying MBNL1 [120] (Table 2). In all cases, splicing correction and reversion of myotonia was obtained in HSA^LR^ mice, showing that the increased expression of MBNL proteins could be an alternative therapeutic option. Interestingly, in PBZ-treated mice, the expression of CELF1 remained unchanged and no splicing correction was found for CELF1-regulated exons. In addition, although PBZ elevated the MBNL1 expression levels in HSA^LR^ mouse muscles, the colocalization of the protein with CUG RNA foci was markedly attenuated, suggesting that PBZ inhibits the interaction between CUG RNA foci and MBNL1 and reduces the ratio of MBNL1 to CUG in the mutant transcript [138]. Amelioration of the DM1-like phenotype by increasing MBNL1 expression was also obtained with miRNA sponge constructs and antagomiRs (cholesterol-2′OMe-ASOs) specific to microRNAs targeting the 3′ UTR of MBNL1/2 mRNAs in a *Drosophila* model of DM1 and in the HSA^LR^ mouse model, respectively [115,116,140,141]. More recently, a peptide-linked blockmiR (Pip9b2-PMO) that binds specifically to the miRNA binding site of the MBNL1 transcript was designed to restrict mis-splicing rescue to disease-relevant alterations, leaving the miRNA available to other targets [117] (Table 2). Furthermore, other drugs such as histone-deacetylase inhibitors induce de-repression of MBNL1 expression in DM1 patient-derived cells [142].

### 4.4. Targeting MBNL:CUG-Repeat Interactions

The recent development of robust high-throughput screens and molecular-design software have facilitated the rapid assessment of hundreds of compounds that disrupt MBNL:CUG binding in vitro. Moreover, expanded CUG repeats have intrinsic biophysical properties favorable to drug development [143]. The rational basis in searching for compounds with therapeutic potential is that the active molecules must specifically interact with the target mutant RNA and release the sequestered MBNL proteins [144]. In addition, these molecules must have a higher affinity for RNA than for DNA, and must show optimal cellular permeability and bioactivity [145,146]. On these bases, a number of groups have pursued the search for new therapeutic molecules. Most candidate compounds compete with MBNL1 for binding to CUG repeats and release the sequestered protein, whereas other molecules unfold or tighten the hairpin structure [147,148,149,150,151,152,153]. The small molecules cugamycin and its more selective and less toxic variant deglyco-cugamycin specifically cleave the expanded CUG repeats, recognizing the tridimensional structure rather than the sequence, and leave short repeats untouched [154,155]. An interesting paper describes the identification of two distinct molecules: the thiophene-containing small molecule that binds MBNL1 in the Zn-finger domain and inhibits the interaction of MBNL1 with its natural pre-mRNA substrates, and a substituted naphthyridine that binds CUG repeats and displaces MBNL1. While the second molecule is effective in reversing DM1-associated splicing alterations, the first molecule that targets MBNL1 causes the dysregulation of alternative splicing, suggesting that MBNL1 is not a suitable therapeutic target for the treatment of DM1 [156]. Potential drug candidates were validated by a variety of functional assays, from in vitro studies to utilization of DM1 animal models. The release of MBNL proteins from CUG repeats restores MBNL activity and rescues splicing defects in DM1 model systems (reviewed in [157]). The widely used rRNA-binding antibiotic erythromycin has been shown to reduce RNA toxicity and improve splicing abnormalities in HSA^LR^ mice by inhibiting the interaction between MBNL1 and CUG expansions [158]. Accordingly, erythromycin is currently being used in a clinical trial (JPRN-jRCT2051190069) in adult DM1 patients [159]. A recent report describes an alternative RNA-binding-protein-mediated strategy to disrupt the MBNL1 interaction with CUG repeats: a truncated MBNL1 (MBNL1∆) protein maintaining CUG-repeat binding but lacking the C-terminal domain that is implicated in splicing activity, cellular localization and oligomerization, was expressed in DM1 patient-derived cells and in HSA^LR^ mice and led to the long-lasting correction of molecular and phenotypical DM1-associated alterations. Interestingly, in contrast with overexpression of the wt MBNL1 protein, the truncated mutant did not produce toxic effects in treated animals [121] (Table 2). Importantly, it should be considered that releasing MBNL proteins from CUG repeats could lead to side effects such as RAN translation from the cytoplasmic expanded transcript, resulting in the production of toxic RAN peptides or, for the drugs that bind CUG repeats with high affinity, a delay in toxic RNA degradation.

### 4.5. Targeting CELF1 and Signaling Molecules Downstream of CUG-Repeat RNA

Other therapeutic strategies have been used to target signaling pathways downstream of CUG-repeat RNA expression, mainly involving CELF1 regulation. PKC inhibitors, reversing CELF1 hyperphosphorylation and upregulation, were shown to rescue some CELF1-dependent splicing defects in DM1 patient-derived fibroblast and myoblast cell lines [160], and to improve contractile dysfunction and mortality in the heart-specific EpA960 mouse model [161]. Additionally, in muscle biopsy samples of DM1 patients and in the HSA^LR^ mouse model showing increased expression of CELF1, reduced cyclin D3 levels and increased levels of glycogen synthase kinase 3 β (GSK3β), which is a known negative regulator of cyclin D3, were detected [162]. The treatment of mice with the GSK3β inhibitors, lithium and 4-benzyl-2-methyl-1,2,4-thiadiazolidine-3,5-dione (TDZD-8), restored the balance of cyclin D3-CELF1 and reversed myotonia and muscle strength in treated HSA^LR^ mice, showing promise for targeting GSK3β as a muscle therapy for DM1 [162]. More recently, an orally available GSK3β inhibitor, Tideglusib (TG), was tested in both the HSA^LR^ and DMSXL mouse models with positive effects on survival, growth, and muscle function. Notably, the correction of GSK3β with TG also reduces the levels of CUG-containing RNA, normalizing a number of CELF1- and MBNL1-regulated mRNA targets [163]. This drug has already been used in a phase-two study in patients with congenital DM1 and improvement of neuromuscular symptoms has been obtained [164]. The AMP-activated protein-kinase (AMPK)/mTOR complex 1 (mTORC1) pathways are affected in HSA^LR^ mice and the activation of AMPK signaling in muscle is impaired under starvation conditions, while mTOR signaling remains active. Short-term treatment with 5-aminoimidazole-4-carboxamide riboside (AICAR), a direct AMPK activator, or with an mTORC1 inhibitor, rapamycin, improved muscle relaxation in mice [165]. Chronic treatment of HSA^LR^ mice using only the AICAR compound led to the correction of muscle histology as well as alternative splicing and toxic transcript aggregation in foci [166]. Apoptosis and autophagy processes have been implicated in the degenerative loss of muscle tissue and regeneration impairment in DM1 [167]. The downregulation of miR-7 was reported in both a *Drosophila* model and in DM1 biopsies [168] and, recently, a link between miR-7 modulation and autophagy and the ubiquitin-proteasome system was demonstrated in DM1 [169]. In addition, it was reported that replenishment of miR-7 improves atrophy-related phenotypes independently of MBNL1, thus suggesting that miR-7 acts downstream of or in parallel to MBNL1 [169]. The biogenesis of miR-7 was shown to be repressed by the RNA-binding protein Musashi-2 (MSI2) and a reduction in MSI2 expression by ASOs enhances miR-7 expression, decreases excessive autophagy and downregulates atrophy-related genes [170]. Altogether, these results suggest that the modulation of miR-7 and/or MSI2 levels could be a therapeutic approach to muscle atrophy in DM1.

### 4.6. Advantages and Limitations of DMPK-Expression Targeting Therapies

The above-described therapeutic molecules have been tested in either in vitro or in vivo models, many in both. Despite the encouraging results obtained in preclinical studies, all of these approaches have some limitations: (i) they require repeated administration; (ii) in general, they only target some aspects/tissues of the disease; (iii) they do not eliminate the disease-causing mutation. Moreover, a considerable challenge associated with recognition specificity and/or selectivity, as well as cellular uptake, still remains for many agents targeting the repeated *DMPK* transcript. Some clinical trials using ASOs on DM1 patients are ongoing and one has been completed. Unfortunately, this first ASO application has obtained limited success [132]. Importantly, among the therapeutic molecules for DM1, some (i.e., erythromycin and tideglusib) are repurposed molecules. Drug repurposing deserves special attention because it offers a number of advantages for new therapies, such as the speed of translation to the clinical setting, safety and cost.

## 5. *DMPK* Gene Editing

### 5.1. Zinc Finger and TALEN Nucleases

The potential of therapeutic CAG/CTG genome editing for monogenic-expansion diseases was initially tested with engineered DNA-cleaving enzymes, including zinc-finger nucleases (ZFNs) and transcription-activator-like effector nucleases (TALENs). The creation of DNA double-strand breaks (DSBs) by ZFNs in regions of CAG/CTG repeats was shown to induce deletions of repeats in yeast [171]. However, the expression of ZFNs targeting CAG-repeat tracts in mammalian cells led to duplications of repeats when breaks were produced, suggesting that targeting the regions flanking the repeats would be preferable [172]. TALEN-induced DSBs were shown to be very efficient at contracting expanded CTG repeats in yeast [173,174]. In human DM1 patient-derived iPSCs, the TALEN system was used to insert of a polyadenylation (polyA) signal upstream of *DMPK* CTG repeats, leading to premature termination of transcription and elimination of toxic mutant transcripts. However, due to TALEN target-sequence constraints, a shorter protein was produced from both wt and mutant *DMPK* edited alleles, and the CTG repeats were retained at the genomic level [43,175] (Table 3). 

### 5.2. CRISPR/Cas9 Gene-Editing Approaches 

More recently, the development and application of the prokaryotic CRISPR (clustered regularly interspaced short palindromic repeats)/Cas9 (CRISPR-associated protein 9) system for genome editing has offered a simpler and more flexible technology [181,182]. The system utilizes the cleaving capacity of the *Streptococcus pyogenes* Cas9 (SpCas9) or the *Staphylococcus aureus* Cas9 (SaCas9) endonucleases, which can be directed to specific DNA or RNA sequences in virtually any genome by engineered single-guide RNAs (sgRNAs) [183,184]. The resultant DNA DSBs are usually repaired by non-homologous end-joining (NHEJ), but other repair mechanisms may also be employed [185,186]. A growing collection of natural and engineered Cas9 homologues and other CRISPR/Cas RNA-guided enzymes is further expanding the manipulation tool in order to modify or interfere with both DNA and RNA [187] (Table 3). In human cells, Cinesi et al. reported that while DSBs induced by either ZFN or Cas9 nuclease within the repeat tract caused instability by inducing both contractions and expansions, the mutant CRISPR/Cas9 D10A nickase mainly induced contractions, independently of single-strand break repair, and no detectable off-target mutations. The authors proposed that DNA gaps lead to contractions and that the type of DNA damage present within the repeat tract dictates the level and the direction of the CAG/CTG-repeat instability [176]. Different editing strategies have been used to contrast the toxic effect of the mutant *DMPK* transcript in DM1: (i) blocking transcription using deactivated Cas9 (dCas9) along with sgRNAs targeting CTG repeats [124]; (ii) degradation of single-stranded CUG-repeat RNA using RNA-targeting Cas9 (RCas9) [177]; (iii) insertion of a premature polyA signal in the *DMPK* gene [178]. In all cases suppression of the mutant RNA transcript, rescue of splicing defects, and reduction in nuclear foci were obtained in DM1 patient-derived cells and iPSCs [188]. In vivo expression of enzymatically inactive Cas9 and RNA-targeting Cas9 was also shown to rescue the muscle phenotype in HSA^LR^ mice [124,125]. While these approaches are very efficient in selectively reducing mutant *DMPK* mRNA, they require persistent expression of the CRISPR elements in order to maintain suppression and do not exclude the pathogenetic impact of CTG-repeat tract at the *DMPK* locus [11,12,13,14]. For therapeutic application, the induction of a permanent correction of the genetic defect would be preferable. To this aim, CRISPR/Cas9-mediated technology was also exploited for excision of DM1 expansion through simultaneous double cuts upstream and downstream of CTG repeats. Successful expansion deletion was obtained using many different cell models, such as DM1 patient-derived immortalized myoblasts and MYOD1-convertible fibroblasts and immortalized myoblasts from DM1 transgenic animals [40,123,179]. Additionally, stem cells such as iPSCs and iPSC-derived skeletal-muscle cells, cardiomyocytes and neural stem cells as well as human embryonic stem cells were used to test the editing of CTG repeats [178,180,188,189]. In all these studies, the removal of the CTG expansion was accompanied by a reduction in nuclear foci and a reversal of aberrant splicing patterns. Repeat deletion and a decrease in ribonuclear foci was also achieved in skeletal muscle of DMSXL mice following injection of rAAV vectors expressing CRISPR/SaCas9 [123]. Interestingly, a recent study described the use of a tetracyclin-repressor-based CRISPR/enhanced SpCas9 (eSpCas9) to obtain a time-restricted and muscle-specific CTG-repeat deletion in DM1 patient-derived myogenic cells and in skeletal muscle of DMSXL mice, highlighting the possibility of exerting temporal control on gene editing [122]. Importantly, transcriptomic, proteomic, and morphological studies performed on DM1 myoblasts subjected to CRISPR/Cas9-mediated excision of the expanded repeats revealed that the reversion of DM1-specific features is maintained over time [190]. In all the above-described studies, delivery of the CRISPR/Cas9-complex components to DM1 mice was achieved through rAAVs that represent, at the moment, the most effective transduction system for therapy in both mouse and human (Table 2). Of note, the recently generated myotropic [191,192] and CNS-tropic AAV variants [193] show enhanced expression in these tissues, holding great potential for gene therapy.

### 5.3. Specificity and Safety of Gene-Editing Approaches

An important concern in using the CRISPR/Cas9 gene-editing approach for DM1 is the occurrence of unintended on-target events during DNA DSB repair and off-target events in undesired loci. While off-target events have rarely been described in published papers, short indels are frequently detected at the joining sites (Table 3). However, these are less likely to be harmful since they occur in a non-coding region of the *DMPK* gene [40,122,179,180]. In contrast, other events such as large deletions and inversions may affect the function of *DMPK* and neighboring genes. On-target deletions following Cas9-induced DSB have been described in different genomic regions and within the CTG repeats, and appear to be cell-context dependent and correlated with the sgRNA target sequence [173,194,195,196]. DSBs elicited by Cas9 nucleases are mainly repaired by the NHEJ, which is a fairly accurate repair system active in all cell-cycle phases including the post-mitotic state. This is important for in vivo CRISPR/Cas9 applications, since most DM1-affected tissues, such as striated muscles and CNS, are post-mitotic. In addition to the efficiency of the DNA-repair system, the cell-cycle state, and the chromatin condition of the target sites, successful and precise editing also depends on the properties of the nuclease and the design of the utilized guide RNAs [197]. Tissue-specific and time-restricted Cas9 activity is a promising tool for reducing the risk of unintended genomic effects [122]. Available software can predict, to some extent, adverse on-target events, and can be used to design accurate guide RNAs [196,198]. Recently, a high-throughput platform using flow-based imaging was developed for the detection of DNA damage in CRISPR/Cas-treated primary stem cells and for the screening of sgRNAs having a single on-target site [199]. Clinical trials relying on the use of genome-editing tools have been initiated for the treatment of both genetic and non-genetic diseases, but not yet for DM1, and have shown promising early results. It is critical to define the potential genotoxic events caused by each specific genome-editing strategy and to develop new tools and define criteria to identify genome-editing-induced events and, importantly, to distinguish them from pre-existing genomic aberrations.

## 6. Concluding Remarks

Although much progress has been made in the treatment of DM1, many issues still remain unsolved. Due to the multisystemic involvement of DM1 disease manifestation, a challenging issue is the efficient delivery of therapeutic molecules to body tissues, particularly to muscle and the CNS, which represent the majority of the body mass. Indeed, the delivery of small molecules such as antisense oligonucleotides or small drugs to these tissues is a major impairment in ongoing clinical trials, while also taking into account that they require repeated administrations. Repurposed drugs have shown good therapeutic properties in DM1 preclinical models, and some of them are being tested in clinical trials. These compounds can be a good temporary solution while waiting for the development of more specific and definitive therapies, since information on methods of delivery and possible side effects are already available. Genome-editing tools hold promise for future therapy and are particularly attractive because they act to permanently remove the cause of the disease. However, they pose a number of questions for application to DM1 patients, mainly concerning the safety and the elicitation of an immune response to CRISPR/Cas9-complex components, particularly when multiple treatments are required [200]. Efficient delivery of the editing molecules via rAAVs with enhanced muscle/nerve-tissue tropism and spatiotemporal control of gene editing through the use of inducible promoters are recently developed tools that can help improve the therapeutic outcome of CRISPR/Cas9-based technologies.

## Figures and Tables

**Table 1:** DM1 mouse models used for studying pathogenetic mechanisms and/or molecular therapies.

**(A) Knockout and Overexpressing Models**												
**Mouse Model**			**Generation Strategy**		**DM1-Like Features**		**Limitations**		**Research Application**		**Ref**	
**DMPK^-/-^**			*Dmpk* KO via replacement of 5′-UTR and exons 1-7 with hygromycin cassette		Late-onset mild myopathy and altered Ca^++^ homeostasis		Mild phenotype; possible confounding insertional effects on flanking genes; mixed genetic background		Relevance of absence of DMPK protein to DM1 phenotype		[52,66]	
**DMPK^-/-^**			*Dmpk* KO via replacement of 5′-UTR and exons 1-7 with neomycin cassette		Late-onset mild myopathy; decreased force generation; altered Na^+^ currents in skeletal muscles; cardiac conduction defects		Mild phenotype; possible confounding insertional effects on flanking genes; mixed genetic background		Relevance of absence of DMPK protein to DM1 phenotype		[51,67]	
**DMPK^-/-^**			*Dmpk* KO via replacement of 5′-UTR and exons 1-7 with hygromycin cassette		No phenotype		Failure to replicate the DM1 phenotype		Relevance of absence of DMPK protein to DM1 phenotype		[53]	
**Mbnl1^ΔE3/ΔE3^**			*Mbnl1* KO via targeted deletion of *Mbnl1* exon 3		Mild myotonia and myopathy (centralized nuclei, split fibers); heart conduction defects; progressive cataracts; AS alterations		Mild muscle phenotype; mild brain alterations; limited spliceopathy		Evaluation of MBNL1 splicing regulation to DM1 phenotype		[56,57]	
**Mbnl2^ΔE2/ΔE2^**			*Mbnl2* KO via targeted deletion of *Mbnl2* exon 2		Development of several CNS alterations (REM sleep propensity, deficit in spatial memory, decreased synaptic plasticity), AS alterations		Failure to replicate the DM1 muscular phenotype		Evaluation of MBNL2 splicing regulation to DM1 phenotype		[55]	
**Mbnl3^ΔE2^**			*Mbnl3* KO via targeted deletion of *Mbnl3* exon 2 (X-linked)		Progressive delay in muscle regeneration; abnormalities in embryonic muscle differentiation leading to neonatal hypotonia		Possible compensation by MBNL3 truncated isoform or other MBNl family members		Evaluation of MBNL3 contribution to DM1 phenotype		[58]	
**Mbnl1^ΔE3/ΔE3^;** **Mbnl2^C/C^;** **Myo-Cre^+/-^**			*Mbnl1* KO; skeletal-muscle specific Cre-mediated *Mbnl2* KO		Small size at birth and skeletal abnormalities; myopathy and severe motor deficits; AS alterations also in brain tissues		High neonatal mortality and reduced lifespan		Evaluation of MBNL1 and MBNL2 contribution to DM1 muscular phenotype		[60]	
**Mbnl1^ΔE3/ΔE3^;** **Mbnl3^ΔE2^**			*Mbnl1* and *Mbnl3* KO via targeted deletion of *Mbnl1* exon 3 and *Mbnl3* exon 2		Myotonia and myopathy; reduction in muscle strength; chloride currents alteration; AS alterations; translation defects		AS alterations similar to *Mbnl1* single knock out; lack of brain alterations		Evaluation of MBNL1 and MBNL3 contribution to DM1 phenotype		[59]	
**Mbnl1^ΔE3/ΔE3^;** **Mbnl2^C/C^;** **Mbnl3^C^;** **Myo-Cre^+/-^**			*Mbnl1* KO; muscle-specific Cre-mediated *Mbnl2* and *Mbnl3* KO		Severe congenital myopathy and spliceopathy, severe respiratory difficulties and muscle wasting in adults; gene expression changes		High neonatal mortality and reduced lifespan		Evaluation of all MBNL proteins loss contribution to DM1 muscular phenotype		[61]	
**MCKCUGBP1**			Insertion of human *CELF1* transgene under striated-muscle-specific MCK mouse promoter		Chains of central nuclei in myofibers, increased NADH reactivity, degenerating fibers and AS alterations		Neonatal lethality in mice expressing high levels of CELF1		Contribution of CELF1 overexpression to DM1 muscular phenotype		[62]	
**TRECUGBP1**			Insertion of Tet-responsive human *CELF1* transgene; heart-specific rtTA expression		Left ventricular systolic dysfunction and dilatation, AS alterations		DM1-like phenotype limited to heart defects		Contribution of CELF1 overexpression to DM1 heart phenotype		[63]	
**TRECUGBP1**			Insertion of Tet-responsive human *CELF1* transgene; skeletal-muscle-specific rtTA expression		Myofibers containing central nuclei, decreased muscle weight, impaired muscle function, AS alterations		DM1-like phenotype limited to skeletal-muscle defects		Contribution of CELF1 overexpression to DM1 skeletal-muscle phenotype		[65]	
**TRECUGBP2**			Insertion of Tet-responsive human *CELF2* transgene; heart-specific rtTA expression		No observed heart pathology; AS alterations similar to those observed in TRECUBP1 mice		Mild heart phenotype		Contribution of CELF2 overexpression to DM1 heart phenotype		[64]	
	**(B) Transgenic Models with Repeat Expansion**											
	**Mouse Model**	**Generation Strategy**		**(CTG)n**		**DM1-Like Features**		**Limitations**		**Research Application**		**Ref**
	**DM200**	Insertion of a Tet-responsive expanded *DMPK* transgene where *DMPK* coding region is replaced by GFP		200		Ribonuclear foci; MBNL1 sequestration; AS alterations; myotonia, progressive cardiac conduction abnormalities		Splicing alterations in the heart have not been described		Study of DM1 phenotype associated with toxic CUG repeats; modeling muscle regeneration; test of therapeutic strategies		[68,69,70]
	**DM300**	Insertion of a 45Kb human genomic fragment containing *DMWD*, *DMPK* and *SIX5* genes from a DM1 patient		~300		Ribonuclear foci (skeletal muscle, heart and brain); myotonia; muscle atrophy; morphological abnormalities; changes in the distribution of MAPT/Tau protein isoform; defect in glucose metabolism		High mortality; mild splicing alterations; intergenerational instability of CTG-repeat numbers		Evaluation of *DMPK* transcript toxicity in different tissues		[71,72]
	**DMSXL**	Insertion of a 45Kb human genomic fragment containing *DMWD*, *DMPK* and *SIX5* genes from a DM1 patient		>1000		Ribonuclear foci; MBNL1 sequestration; AS alterations; deficits in motor performance; behavioral abnormalities; synaptic dysfunction; inhibition of exploratory activity and cerebellar glial dysfunction		High mortality; severe body-weight reduction; interindividual variability; decreased transgene expression with aging; mild muscular phenotype		Evaluation of *DMPK* transcript toxicity in different tissues and in multiple brain cell types; test of therapeutic strategies		[23,73,74]
	**HSA^LR^**	Insertion of the human skeletal actin (*HSA*) gene including CTG repeats in the 3’ UTR		~250		Ribonuclear foci; AS alterations; myotonia and muscle histopathology abnormalities (increase in central nuclei and variability in fiber size) after six months of age		Limited to skeletal muscle; does not contain *DMPK* gene sequence; absence of muscle weakness		Investigation of expanded-CUG-repeat toxicity in muscle fibers; test of therapeutic strategies		[75,76]
	**LC15**	Insertion of CTG expanded *DMPK* 3’ UTR downstream Luciferase gene driven by CMV-βA promoter		250–400		Ribonuclear foci, AS alteration and MBNL2 upregulation in the heart; reduced Na^+^ and K^+^ channel activity; ventricular arrhythmias		DM1-like phenotype limited to heart defects		Evaluation of biophysical mechanisms reproducing DM1-like electrocardiograph abnormalities		[77]
	**EpA960/** **𝛼** **-MHC-Cre**	Insertion of CTG expanded *DMPK* exon 15 transgene containing Cre-responsive loxP sequences; heart-specific myosin Cre expression		960 (CTCGA-interrupted)		Ribonuclear foci; MBNL1 sequestration; CELF1 protein upregulation; AS alterations; cardiomyopathy, arrhythmias; systolic and diastolic dysfunction		Does not reproduce CTG-repeat continuity; mouse model no longer available		Evaluation of *DMPK* transcript toxicity and CELF1 overexpression in heart tissue		[78]
	**EpA960/** **HSA-Cre**	Insertion of CTG expanded *DMPK* exon 15 transgene containing Cre-responsive loxP sequence; skeletal-muscle-specific Cre expression		960 (CTCGA-interrupted)		Ribonuclear foci; MBNL1 sequestration; CELF1 protein upregulation; AS defects; myotonia and progressive muscle wasting, deficits in muscle performance and histopathological abnormalities		Does not reproduce CTG-repeat continuity; mouse model no longer available		Evaluation of *DMPK* transcript toxicity and CELF1 overexpression in skeletal tissue		[79]
	**EpA960/** **CamKII-Cre**	Insertion of CTG expanded *DMPK* exon 15 transgene containing Cre-responsive loxP sequence; brain-specific Cre expression		960 (CTCGA-interrupted)		Ribonuclear foci; MBNL1 sequestration; AS alterations; learning disability; neurotransmission dysfunction; brain atrophy and aging		Does not reproduce CTG-repeat continuity; mouse model no longer available		Identify mechanisms involved in CTG-dependent neuronal degeneration		[80]
	**TREDT960I/** **𝛼** **-MHC-rtTA**	Insertion of Tet-responsive expanded *DMPK* exons 11–15 transgene; heart-specific rtTA expression		960 (CTCGA-interrupted)		Ribonuclear foci; MBNL1 sequestration; CELF1 protein upregulation; AS alterations ; arrhythmias		Does not reproduce CTG-repeat continuity		Study of alteration of ion transport and action potential in cardiomyocytes expressing toxic CUG		[81,82]
	**TREDT960I/** **MDAF-rtTA**	Insertion of Tet-responsive expanded *DMPK* exons 11–15 transgene; skeletal-muscle-specific rtTA expression		960 (CTCGA-interrupted)		Ribonuclear foci; MBNL1 sequestration; CELF1 protein upregulation; AS alterations; muscle wasting and myopathy		Does not reproduce CTG-repeat continuity		Study the mechanisms of CUG-repeat-induced muscle tissue loss		[83]

Abbreviations: AS = alternative splicing; ChP = brain choroid plexus; CMVβA = cytomegalovirus enhancer/β-actin; GFP = green fluorescent protein; KO = knockout; MDAF = expression vector carrying regulatory sequences for the rat myosin light chain 1/3 gene; MHC = myosin heavy chain; Myo = myogenin; NADH = nicotinamide adenine dinucleotide; polyA = polyadenylation; rtTA = reverse tet transactivator.

**Table 2:** Nucleic-acid-based molecules in preclinical studies and clinical trials for DM1.

Molecule Class	Target	Therapeutic Molecule	Mechanism	DDS	Admin. Route	Study Phase	Ref
**ASOs**	*DMPK* CUGexp	PMO-CAG25, 2′-OMe-CAG, LNA-CAG mixmers, all-LNA-CAG	MBNL1 binding block	Naked	IM	Preclinical	[92,103,104,105]
	*DMPK* CUGexp	PPMO-B, PPMO-K; Pip6a-PMO	MBNL1 binding block	CPP-conj	IM, IV	Preclinical	[106,107]
	*DMPK* CUGexp	miniPEG-γ PNA	MBNL1 binding block	Polymer-conj	SC	Preclinical	[108,109]
	*DMPK* 3′UTR	MOE gapmers, c-Et gapmers, LNA gapmers	*DMPK* mRNA degradation	Naked	IM, SC, ICV	Preclinical	[54,70,110,111,112]
	*DMPK* CUGexp	LNA gapmers, MOE gapmers	Mutated *DMPK* mRNA degradation	Naked	IM	Preclinical	[113]
	*DMPK* 3′UTR	IONIS-DMPKRx	*DMPK* mRNA degradation	Naked	SC	Clinical(completed)	[114]
	*DMPK* 3′UTR	palmitoyl-c-Et gapmers	*DMPK* mRNA degradation	Lipid-conj	SC	Preclinical	[69]
	miRNAs targeting *Mbnl1* mRNA	cholesterol-2′OMe-ASOs	AntagomiR	Lipid-conj	SC, IV	Preclinical	[115,116]
	*Mbnl1* 3′UTR	Pip9b2-PMO	BlockmiR	CPP-conj	IV	Preclinical	[117]
**siRNA**	*DMPK* CUGexp	siRNA-CAG	Mutated *DMPK* mRNA degradation	Nacked	IM	Preclinical	[118]
	*DMPK* mRNA	AOC 1001	*DMPK* mRNA degradation	Ab-conj	IV	Clinical (recruiting)	[119]
**rAAV**	*DMPK* downstream pathway	MBNL1	MBNL1 overexpression	rAAV1	IM	Preclinical	[120]
	*DMPK* downstream pathway	MBNL1	Competition for CUGexp interaction	rAAV9	IM	Preclinical	[121]
	*DMPK* CTG spanning region	Sa/eSpCas9-sgRNAs	CTGexp removal	rAAV9	IM	Preclinical	[122,123]
	*DMPK* CTGexp	dSaCas9-sgRNA	Transcription inhibition	rAAV6, rAAV9	IV	Preclinical	[124]
	*DMPK* CUGexp	RCas9-sgRNA	*DMPK* mRNA degradation	rAAV9	IV, TA	Preclinical	[125]

Abbreviations: Ab-conj = antibody-conjugated; Admin. Route = administration route; CPP = cell-penetrating peptide; CTGexp = CTG expansion; CUGexp = CUG expansion; DDS = drug delivery system; dSaCas9 = deactivated *Staphylococcus aureus* Cas9; ICV = intracerebroventricular; IM = intramuscular; IV = intravenous; Naked = not conjugated ASOs; rAAV = recombinant adeno-associated virus; RCas9 = RNA targeting Cas9; RO = retro orbital; eSpCas9-sgRNAs = enhanced *Streptococcus pyogenes Cas9*-single guide.

**Table 3:** Gene-editing strategies.

Nuclease	Mechanism	Effect	DM1 Model	Advantages	Limitations	Ref
**ZNF**	Induction of DNA double strand breaks at CAG/CTG repeats	Repeat contractions	Yeast cells carrying CTG repeats	Permanent reduction in CTG repeats; good cleavage efficiency	Repeat rearrangements	[171]
**ZNF**	Induction of DNA double strand breaks at CAG/CTG repeats	Repeat contractions and duplications	Mammalian cells carrying CTG repeats	Permanent reduction in CTG repeats; good cleavage efficiency	Repeat duplications	[172]
**TALEN**	Induction of DNA double strand breaks at CAG/CTG repeats	Repeat contractions	Yeast cells carrying CTG repeats	Permanent reduction in CTG repeats; good cleavage efficiency, no mutations	Application limited to yeast cells	[173,174]
**TALEN**	Insertion of a polyA signal upstream CTG repeats	Production of shorter *DMPK* transcripts (no CUG)	DM1-patient-derived iPSCs	Elimination of toxic CUG repeats from *DMPK* transcript	Production of truncated DMPK protein; retention of CTG at *DMPK* locus	[43,175]
**SpCas9 D10A**	Induction of DNA single strand breaks at CAG/CTG repeats	Repeat contractions	Human cells carrying CAG/CTG repeats	Permanent reduction in CTG repeats	Cell-type dependent efficiency	[176]
**dCas9**	Block DNA transcription at CTG repeats	*DMPK* transcription inhibition	DM1-patient-derived cells; HSA^LR^ mice	Suppression of CUG-repeat-transcript production	Decreased DMPK protein production; retention of CTG at *DMPK* locus; need of repeated treatment	[124]
**RCas9**	Cleaving single-strand RNA at CUG repeats	CUG-repeated transcript degradation	DM1-patient-derived cells; HSA^LR^ mice	Elimination of CUG-repeat transcript	Decreased DMPK protein production; retention of CTG at *DMPK* locus; need of repeated treatment	[125,177]
**SpCas9** **D10A**	Insertion of a polyA signal upstream CTG repeats	Production of shorter *DMPK* transcripts (no CUG)	DM1-patient-derived iPSCs	Elimination of toxic CUG repeats from *DMPK* transcript	Production of truncated DMPK protein; retention of CTG at *DMPK* locus	[178]
**SpCas9** **or SaCas9**	Induction of two DNA double strand breaks at CTG-repeats flanking regions	Deletion of CTG expanded region	DM1-patient-derived iPSCs	Permanent elimination of toxic CTG repeats; no off-targets	Low efficiency using SpCas9; higher efficiency but frequent inversions using SaCas9	[178]
**SpCas9**	Induction of two DNA double strand breaks at CTG-repeats flanking regions	Deletion of CTG expanded region	DM1-mouse-derived myoblasts; DM1-patient derived myoblasts	Permanent elimination of CTG repeats; no off-targets	On-target indels, inversions, large deletions	[179]
**SpCas9**	Induction of two DNA double strand breaks at CTG-repeats flanking regions	Deletion of CTG expanded region	DM1-patient-derived MYOD1-converted fibroblasts	Permanent elimination of CTG repeats; no off-targets	On-target indels, inversions	[40]
**SpCas9**	Induction of two DNA double strand breaks at CTG-repeats flanking regions	Deletion of CTG expanded region	DM1-patient-derived primary myoblasts; DM1-patient-derived iPSCs	Permanent elimination of CTG repeats; good editing efficiency in iPSCs; no off-targets	On-target indels, partial deletions	[180]
**SaCas9**	Induction of two DNA double strand breaks at CTG-repeats flanking regions	Deletion of CTG expanded region	DM1-patient-derived myoblasts; DMSXL mice	Permanent elimination of CTG repeats; good editing efficiency in DM1 cells; no off-targets	On-target indels; low editing efficiency in mice skeletal muscle	[123]
**eSpCas9**	Induction of two DNA double strand breaks at CTG-repeats flanking regions	Inducible deletion of CTG expanded region	DM1-patient-derived MYOD1-converted fibroblasts; DMSXL mice	Permanent elimination of CTG repeats; well-regulated editing induction; no off-targets	On-target indels, inversions, large deletions	[122]

Abbreviations. dCas9 = deactivated Cas9; eSpCas9 = enhanced *Streptococcus pyogenes* Cas9; RCas9 = RNA-targeting Cas9; SaCas9 = *Staphylococcus aureus* Cas9; SpCas9 = *Streptococcus pyogenes* Cas9; SpCas9 D10A = SpCas9 nickase.
